# Treatment of Atypical Bifid Mandibular Condyle Associated with Ankylosis of the Temporomandibular Joint

**DOI:** 10.1155/2019/6372897

**Published:** 2019-01-10

**Authors:** Katheleen Miranda, André Sander Carneiro, Jennifer Tsi Gerber, Suyany Gabriely Weiss, Leandro Eduardo Klüppel, Rafaela Scariot

**Affiliations:** ^1^School of Health Science, Positivo University, Curitiba, Brazil; ^2^School of Health Science, Federal University of Paraná, Curitiba, Brazil

## Abstract

**Introduction:**

The bifid mandibular condyle (BMC) is an unusual temporomandibular joint (TMJ) disorder with controversial etiology. The association of this entity with ankylosis is rare.

**Objective:**

The objective of the present study is to report a case of BMC with associated TMJ ankylosis in a patient with no history of trauma and/or infection.

**Case Report:**

A 17-year-old male patient sought care reporting pain on the right TMJ region and mastication difficulty due to a severe limitation of mouth opening. In the clinic and imaging examinations, a 15 mm mouth opening and BMC associated with ankylotic mass of the right TMJ were observed, besides a facial asymmetry with chin deviation to the right. The proposed treatment plan was condylectomy on the right side, bilateral coronectomy, and genioplasty, so the chin lateral deviation could be corrected, under general anesthesia. The patient remains under clinical and imaging follow-up of two years with functional stability and no signs of relapse of the ankylosis.

**Conclusion:**

The association of BMC with ankylosis is an atypical entity which must be diagnosed and treated early to prevent aesthetic and functional damages to the patient.

## 1. Introduction

Bifid mandibular condyle (BMC) is an anomaly characterized by a division of the mandibular head [1]. It was described for the first time by Hrdlicka in 1941, in 21 cases of a series of skull specimens. Sicher, in 1948, was the first researcher to report a case of BMC in a living person [2, 3]. Morphologically, a BMC may be restricted to a delicate notching on the condyle or extended as a complete lobulation of the condyles. Extensive division might result in two heads while in cases less completed, the heads are separated simply by a shallow groove [4].

The etiology of this entity is still controversial, even though two main theories have been discussed: traumatic origin and developmental anomaly [3]. Endocrine disorders, deficiency of some nutrients, irradiation, infection, ankylosis, and genetic factors are also cited as the potential cause for BMC induction [5, 6]. Its prevalence is still not clear [3]. The majority of studies sustains that there is no preference when it comes to sex or race and that it is usually unilateral [7]. It is often asymptomatic, being a radiological finding. However, when symptomatic, it may be associated with pain, limitation of mouth opening, ankylosis, facial asymmetry, and swelling in the affected region. Many types of temporomandibular joint (TMJ) imaging methods are adopted for different diagnostic purposes, including conventional radiography, computerized tomography (CT), MRI, ultrasonography, and cone beam CT scan [8].

The association of BMC with TMJ ankylosis is even more unusual, with very few cases reported in literature [9]. This change may cause a deformity of the articular fossa, compromising functions such as the speech, mastication, and limitation of mouth opening [10]. The BMC proper treatment depends on the symptomatology. Patients with associated articular ankylosis might need surgical condylectomy or arthroplasty [9].

Therefore, the objective of this study is to report a case of BMC with associated TMJ ankylosis in a patient with no history of trauma or previous ear infection, as well as its treatment alternatives.

## 2. Case Report

**Figure 1:**
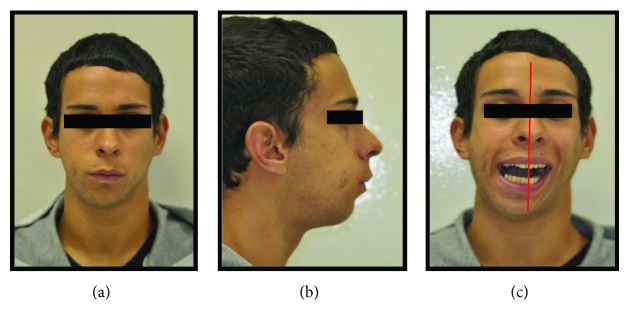
(a), (b), and (c) Preoperative pictures with front and lateral views emphasizing anteroposterior deficiency of the jaw and chin, mouth opening limitation, and chin deviation to the right.

**Figure 2:**
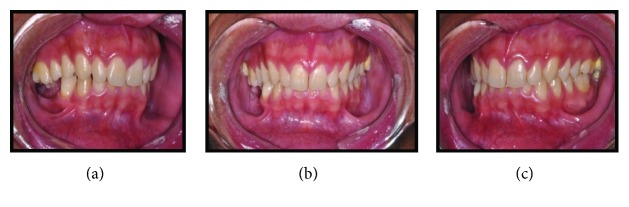
(a), (b), and (c) Preoperative intraoral pictures emphasizing deep bite and teeth absence on the right side.

Written informed consent was obtained from the patient for publication of this case report and accompanying images. Patient D.G.B., a 17-year-old male, with no systemic changes, sought the services of Maxillofacial Surgery and Traumatology of Positivo University, Curitiba-PR, complaining of severe mouth opening limitation and pain on the right TMJ region. In the anamnesis, there was no record of trauma or ear infection during his childhood. At the clinical exam, the mouth opening limitation (15 mm) and a mandibular deviation to the right during the opening movement were verified. In addition, it was possible to observe a chin deviation to the right, associated with anteroposterior deficiency of the jaw and chin (Figure 1). The patient had a deep bite and teeth absence in the right posterior region of the mandible (Figure 2).

**Figure 3:**
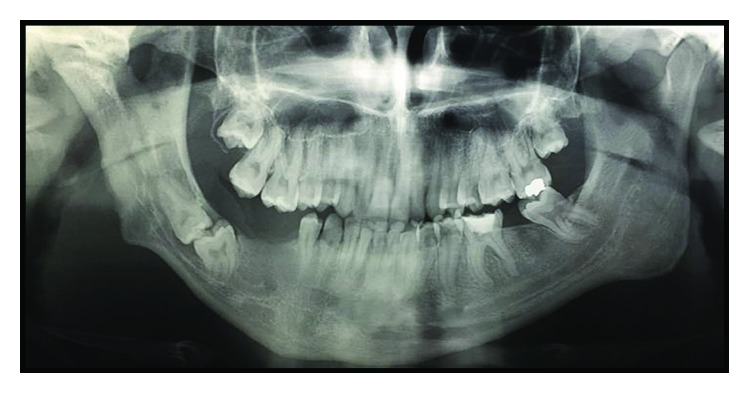
Preoperative panoramic radiography showing altered condylar morphology on the right side, mandibular asymmetry, and impaction of the teeth 47 and 48.

**Figure 4:**
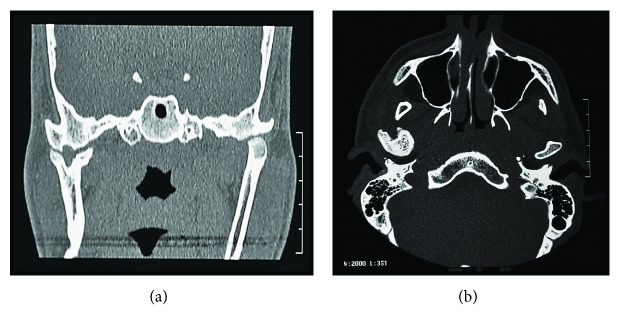
(a) Coronal view of the CT scan emphasizing the bifid mandibular condyle in the laterolateral direction. (b) Axial view of the CT scan showing ankylosing mass.

Imaging exams were requested—panoramic radiography and CT scan—in which could be observed altered condylar morphology on the right side, a characteristic of BMC, associated with TMJ ankylosis. Moreover, a chin deviation to the right and the impaction of the elements 47 and 48, in an unfavorable position and angulation, were observed (Figures 3 and 4).

Based on this information, the proposed treatment plan was condylectomy on the right side and the removal of the bilateral coronoid process in order to detach the temporal muscle fibers and assist with the mouth opening. In addition, in the same surgical time, a genioplasty to the advancement of 10 mm and correction of the lateral chin deviation of 3 mm to the left was suggested. Due to the unfavorable position and absence of associated symptomatology to the impacted teeth 47 and 48, it was chosen not to remove these teeth at the time and have a clinic and radiographic follow-up.

**Figure 5:**
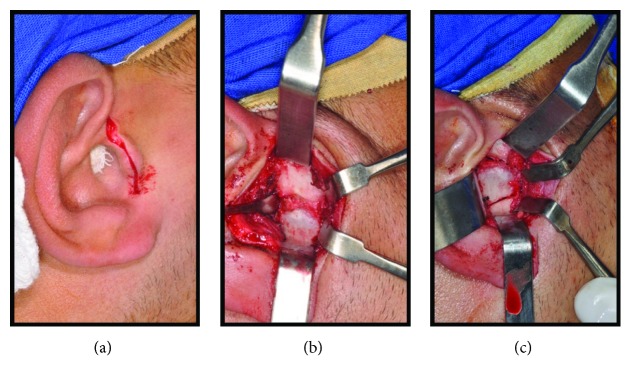
(a), (b), and (c) Transoperative pictures showing the endaural access and the exposure of the ankylosing mass.

The procedure was performed under general anesthesia through a nasotracheal intubation. An extraoral cleansing with iodine 10% with surfactant and intraoral cleansing with topical iodine 10% were conducted. The surgical access chosen was endaural, in order to leave the most imperceptible scar possible. A dissection of the tissues and exposure of the intra-articular spaces were performed with condylectomy associated with the removal of the ankylosing mass with no interposition of tissue or material (Figure 5).

**Figure 6:**
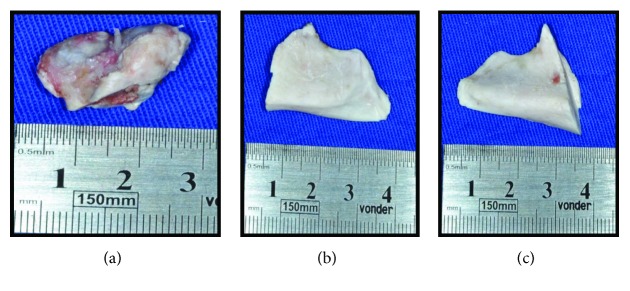
(a), (b), and (c) Surgical removal of the condylar process and bilateral coronoid process.

According to Sawhney's ankylosis classification, the mass found corresponds to type I, when the condyle is medially angled and associated with a deformed joint fossa along with a mild to moderate amount of new bone formation and fibrosis. This can be seen in the clinical picture (Figure 6(a)).

**Figure 7:**
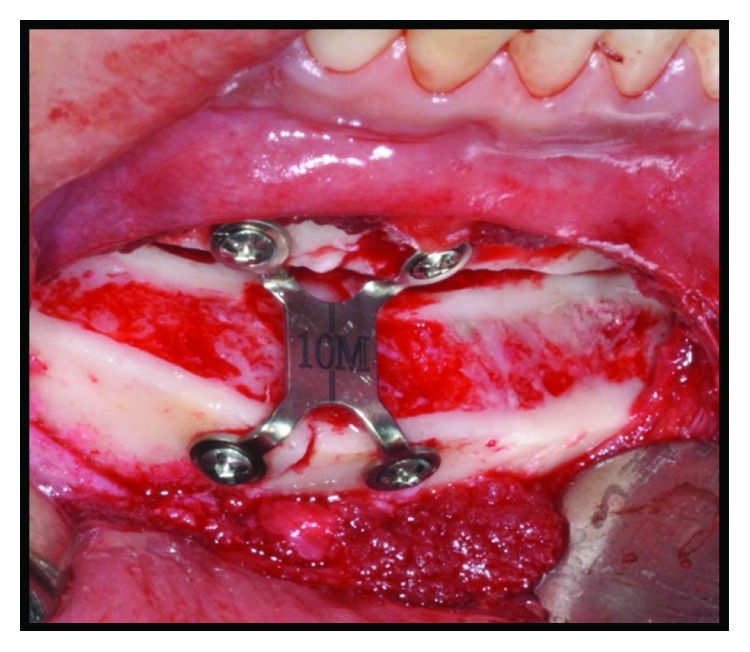
Stable internal fixation of the chin with a chin plate of 10 mm from the 2.0 mm system.

At the same time, a bilateral coronoidectomy and genioplasty were conducted. On the right side, the extraoral access was used to remove the coronoid process, while on the left side, an intraoral access was performed, at the bottom of the vestibule for the exposure and removal of the coronoid process. We chose to remove the right coronoid process to extraoral access only to use the same access used to perform the condylectomy. The stable internal fixation of the chin was conducted with a chin plate of 10 mm and four 2.0 screws (Figures 6 and 7).

**Figure 8:**
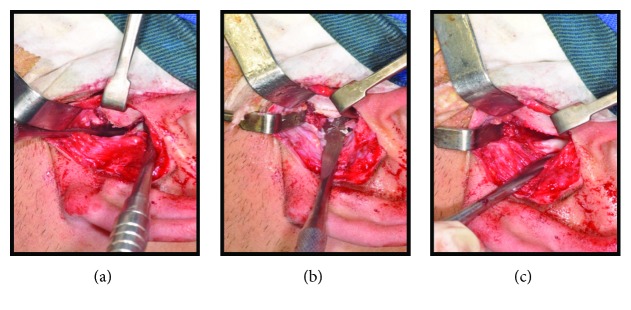
(a), (b), and (c) Transoperative pictures emphasizing the eminectomy on the left side.

**Figure 9:**
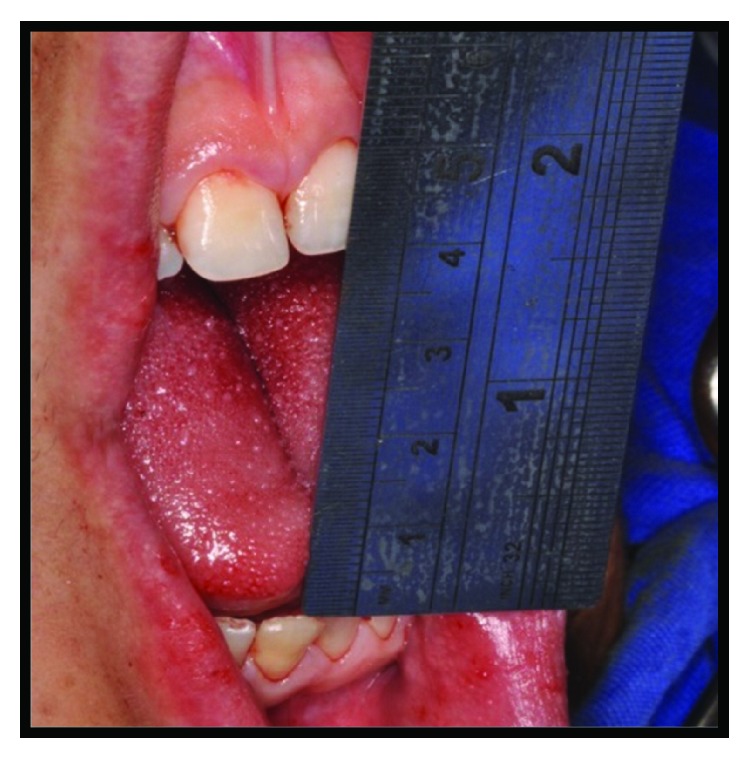
Mouth opening of 39 mm in the transoperative.

At the transoperative, it was observed that the mouth opening was still not satisfactory. For that reason, besides the proposed treatment, an eminectomy on the left side in order to assist with the patient mouth opening was performed (Figure 8). After that, a 39 mm opening in the transoperative was achieved (Figure 9).

**Figure 10:**
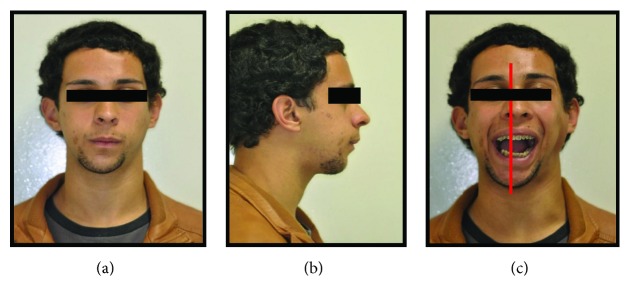
(a), (b), and (c) Postsurgery pictures after seven days emphasizing a more harmonic facial profile and improvement of the mouth opening and the mandibular deviation.

**Figure 11:**
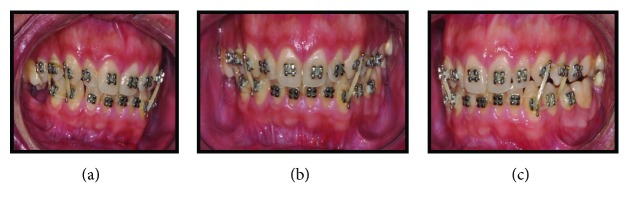
(a), (b), and (c) Intraoral pictures showing occlusion guided with elastic bands.

**Figure 12:**
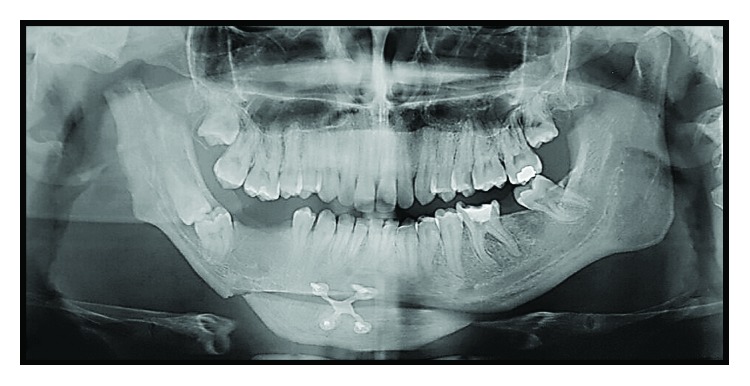
Panoramic radiography after seven days emphasizing the stable internal fixation of the chin and bone gap on the right side of the TMJ region.

**Figure 13:**
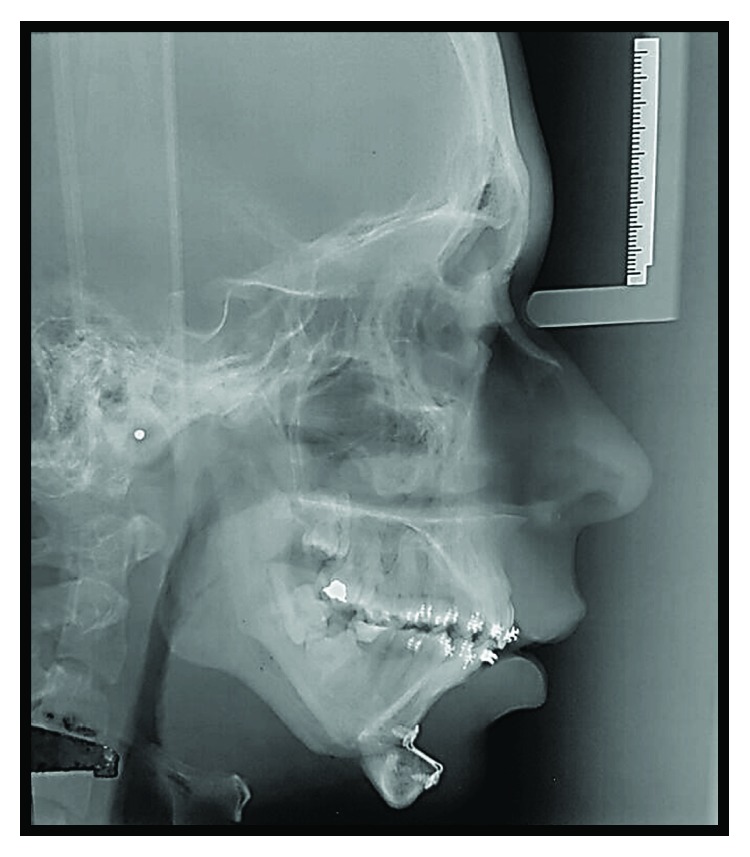
Profile teleradiograph after seven days showing a better facial profile with chin advancement.

At the immediate postsurgery, the patient evolved with a slight anterior open bite, compatible with intra-articular swelling. Therefore, orthodontic brackets were installed so the occlusion could be guided with elastic bands. The patient continued with the follow-ups, with a mouth opening of 22 mm, no pain, and a satisfactory aesthetic and functional result (Figures 10 and 11). Seven days after the surgery, the patient performed a panoramic radiography, and the picture emphasized a good stability of the fixation and the bone gap on the right TMJ (Figures 12 and 13).

**Figure 14:**
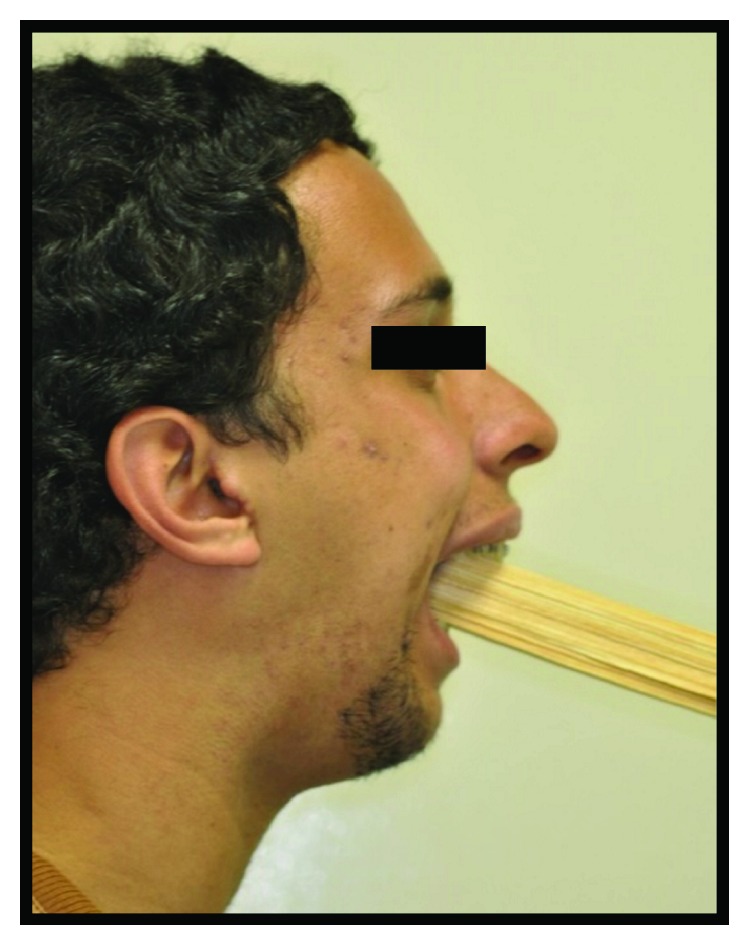
Physiotherapy with 15 wooden spatulas after 15 days of postsurgery.

**Figure 15:**
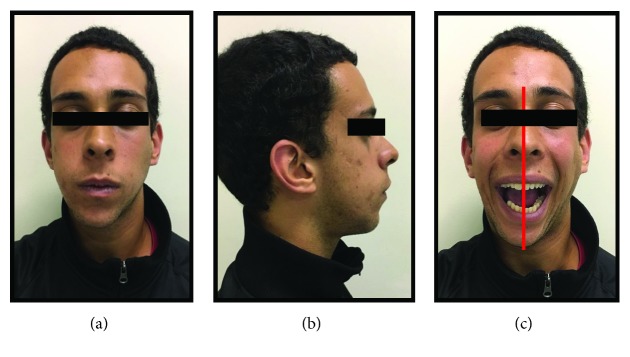
(a), (b), and (c) Postsurgery pictures after two years emphasizing the improvement in the facial harmony, mouth opening, and chin deviation.

**Figure 16:**
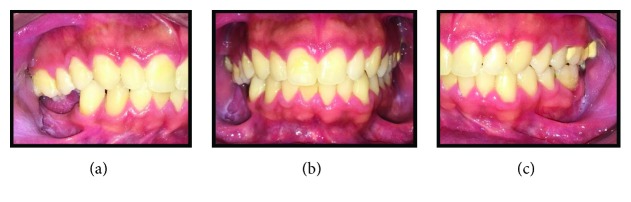
(a), (b), and (c) Intraoral pictures after two years showing stability in the occlusion.

**Figure 17:**
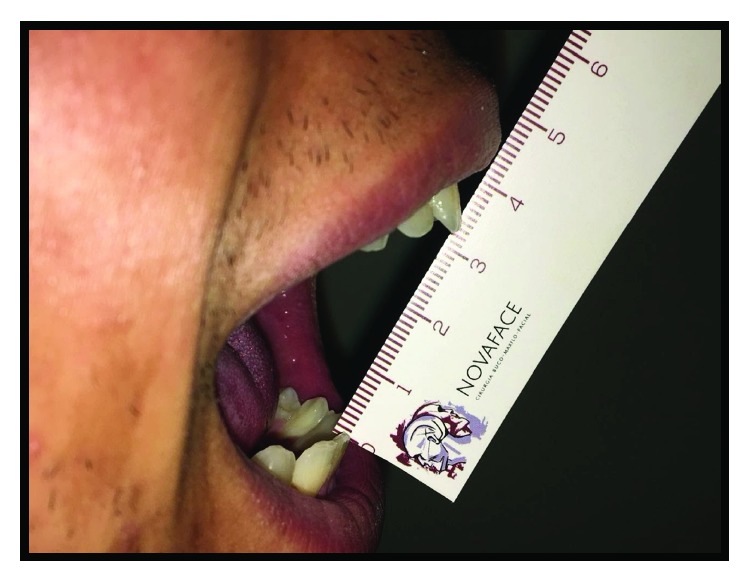
Picture emphasizing the mouth opening of 32 mm after two years of postsurgery.

**Figure 18:**
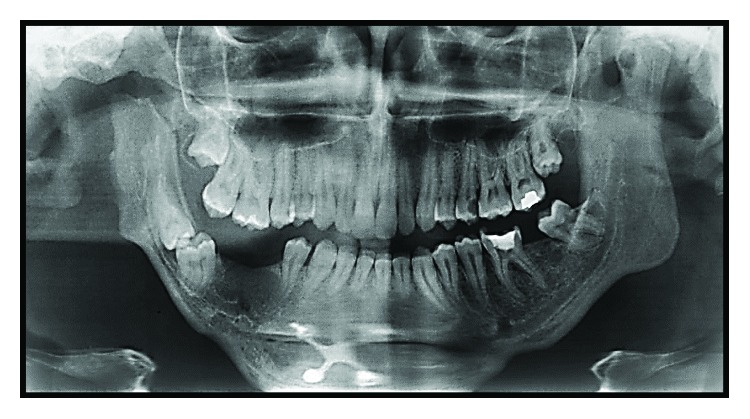
Panoramic radiography after two years emphasizing stability in the chin fixation and with no signs of ankylosis relapse.

**Figure 19:**
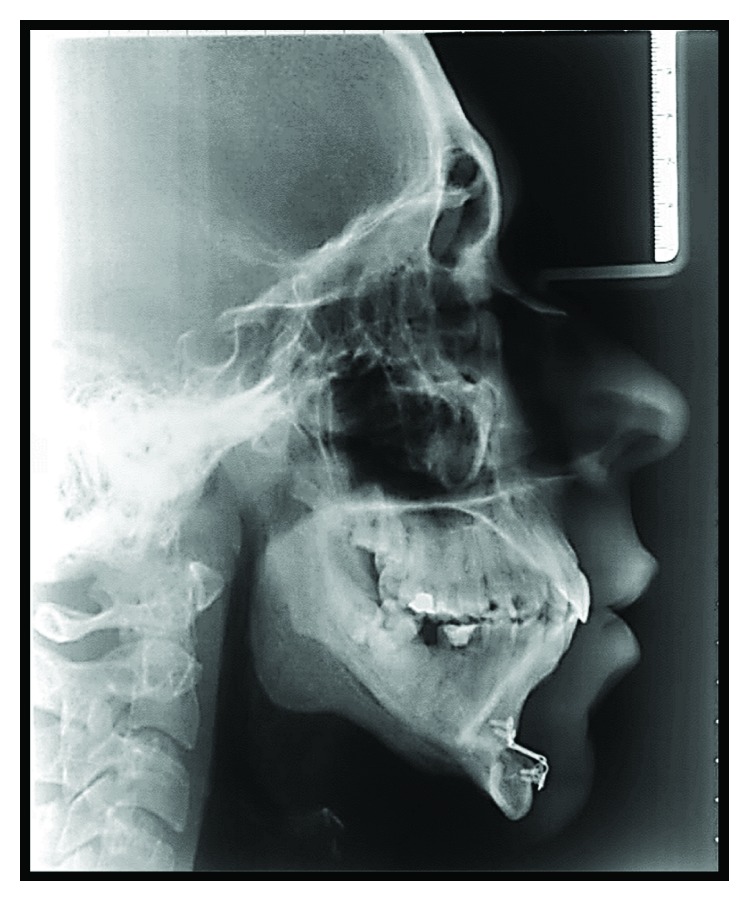
Profile teleradiograph after two years showing stability in the fixation and improvement of the facial profile.

The patient was submitted to physiotherapy sessions with wooden spatulas, and after 30 days of postsurgery, he removed the orthodontic brackets, due to the stability of the occlusion (Figure 14). Since then, the patient remained in clinical and radiographic follow-up of two years, having no complaints and with a mouth opening of 35 mm (Figures 15, 16, 17, 18, and 19).

## 3. Discussion

Bifid mandibular condyle is an unusual condition, even though more cases are being reported as a result of enhanced imaging techniques [6, 7, 9, 11]. Its etiology is still contested. Most studies believe that this condition is associated with a trauma during childhood. However, there are theories about the persistency of fibrovascular septa during the formation of the mandibular condyle, teratogenic embryopathy, unusual insertion of muscle, endocrine disorders, and infection, which might explain the formation of BMC [5, 6, 9, 12]. In addition, the TMJ ankylosis may cause the development of BMC [2]. In a retrospective study, Rehman et al. [6] reported ten cases of BMC in 37 patients with TMJ ankylosis. Out of these ten cases, nine were posttraumatic and one was postinfectious. In the present case, neither the patient nor his family remembered any trauma or infection during his childhood.

Bifid mandibular condyle is often unilateral, happening twice as much on the left side [7, 13]. In the literature, there is not a predilection when it comes to race or age. In a study reported by Loh and Yeo [14], most of the patients were over 20 years old. It is usually a radiographic finding. However, when symptomatic, it may be associated with pain, swelling, asymmetries, and mouth opening limitation [6]. Cho and Jung [15] examined 3046 asymptomatic patients and 4378 patients with symptoms of TMJ dysfunction. They detected 15 (0.49%) cases of BMC in asymptomatic patients and 22 (0.50%) in symptomatic patients. There were no significant differences related to gender or to the affected side or any association with the symptoms in this study. Our report is about a 19-year-old male patient with BMC associated with TMJ ankylosis on the right side, with painful symptomatology and severe limitation of mouth opening, which is different from the usual cases, making this one atypical.

The most common radiographic image used in a diagnosis is the panoramic view, and it is used in most routine dental procedures [8]. However, it is known that the CT scan introduced a new image category, being increasingly used to evaluate the morphology of the mandibular condyle [16, 17]. The most usual imaging appearance is an anterior and a posterior head, separated by a shallow groove, although they can be oriented mediolaterally [3]. The TMJ ankylosis radiological aspect is more variable. There may be a deformity, with a complete loss of joint space and an unusual bone formation around the joint, or even a reduction of the joint space without complete obliteration, suggesting fibrous ankylosis [9, 18]. In this case report, the mandibular condyle was oriented mediolaterally.

The ideal treatment of these conditions depends on the clinical picture. Asymptomatic cases do not need any type of intervention [19]. When related to the ankylosis, the main objective of the treatment is to restore the function [11]. In these cases, Manganello-Souza and Mariani [10] described three basic techniques for the surgical correction of the temporomandibular ankylosis: gap arthroplasty (GA), interpositional arthroplasty (IA), and TMJ reconstruction.

Total joint replacement surgery is the most invasive treatment for TMJ disease. The goal of the procedure is to reduce pain, improve mouth opening, and correct occlusal changes and facial deformities [20]. Gold standard in treatment of this case is TMJ prosthesis, despite the young age of the patient, but this technique is expensive and not performed in all departments. Another option is to reconstruct the condyle with bone (rib) and interposition of fat, but the results are unpredictable.

In the present case, GA was the chosen technique. The GA has some advantages, such as the simple execution, less transoperative time, and low cost [21]. However, some authors have been associating it to a higher ankylosis recurrence rate [22]. A retrospective study designed by Danda et al. [23] could not find significant differences related to the mouth opening and the incidence of recurrence among the patients treated with GA and IA and concluded that the treatment's success depended on the patient's cooperation, active physiotherapy, and regular monitoring. Among the biological and nonbiological materials which can be used to the interposition after the GA are the remnant of the temporal muscle, skin, fat, cartilage, and silicon [22]. In this case report, the patient is in a two-year follow-up of gap arthroplasty, with no signs of recurrence of the ankylosis and with procedure stability. Any material since the patient presented an important gap was not lodged.

The GA is often combined with ipsilateral coronoidectomy because the prolonged immobility of the jaw, due to the ankylosis, usually results in coronoid hyperplasia. A contralateral coronoidectomy may be needed to facilitate the mouth opening [24], like it was performed in this case. Manganello-Souza and Mariani [10] reported 14 cases of TMJ ankylosis. Among the bilateral cases, five were submitted to bilateral coronoidectomy and one to unilateral coronoidectomy, while ipsilateral coronoidectomy was conducted in four of the eight unilateral cases. Moreover, the articular eminence has an important role in the TMJ's biomechanics, and it acts as an anterior barrier for the head of the mandible during the opening movement [25]. In this case report, after the gap arthroplasty and bilateral coronoidectomy, an eminectomy on the left side in order to increase the patient's mouth opening was performed.

It is known that the best treatment for the correction of the anteroposterior deficiency of the jaw is the mandibular advancement through orthognathic surgery. The most commonly performed technique for this condition is sagittal osteotomy of the mandibular ramus. However, some studies argue that the mandibular ramus in patients with ankylosis and with skeletal deformities usually has severe anatomic variations that may complicate or even prevent the sagittal split of the ramus [26]. For these cases, some authors suggest inverted L osteotomy to extend the mandibular ramus and body at the same time [26–28]. In this case report, despite the patient's anteroposterior deficiency, any type of osteotomy was not performed due to the inappropriate positioning of the teeth 47 and 48 and because that was not the patient's complaint. Therefore, it was chosen to work with advancement genioplasty to aesthetically soften the jaw and chin anteroposterior deficiency.

## 4. Conclusion

The association of BMC and ankylosis is an atypical condition that might compromise the patient's function and quality of life. The treatment variations must take into consideration the associated symptoms, the age, and the patient's cooperation.
